# Targeting Endothelial Adhesion Molecule Transcription for Treatment of Inflammatory Disease: A Proof-of-Concept Study

**DOI:** 10.1155/2016/7945848

**Published:** 2016-05-16

**Authors:** Liam M. Ashander, Binoy Appukuttan, Yuefang Ma, Dione Gardner-Stephen, Justine R. Smith

**Affiliations:** Eye & Vision Health, Flinders University School of Medicine, Flinders Medical Centre, Flinders Drive, Bedford Park, SA 5042, Australia

## Abstract

Targeting the endothelial adhesion molecules that control leukocyte trafficking into a tissue has been explored as a biological therapy for inflammatory diseases. However, these molecules also participate in leukocyte migration for immune surveillance, and inhibiting the physiological level of an adhesion molecule might promote infection or malignancy. We explored the concept of targeting endothelial adhesion molecule transcription during inflammation in a human system. Intercellular adhesion molecule 1 (ICAM-1) mediates leukocyte migration across the retinal endothelium in noninfectious posterior uveitis. We observed an increase in the transcription factor, nuclear factor of kappa light polypeptide gene enhancer in B-cells 1 (NF-*κ*B1), in parallel with ICAM-1, in human retinal endothelial cells treated with tumor necrosis factor-alpha (TNF-*α*), and identified putative binding sites for NF-*κ*B1 within the* ICAM-1* regulatory region. We targeted induced NF-*κ*B1 expression in endothelial cells with small interfering (si)RNA. Knockdown of NF-*κ*B1 significantly decreased cell surface expression of ICAM-1 protein induced by TNF-*α* but did not reduce constitutive ICAM-1 expression. Consistently, NF-*κ*B1 knockdown significantly reduced leukocyte binding to cell monolayers in the presence of TNF-*α* but did not impact baseline binding. Findings of this proof-of-concept study indicate that induced transcription of endothelial adhesion molecules might be targeted therapeutically for inflammatory disease in humans.

## 1. Introduction

Inflammatory diseases are characterized by the accumulation of leukocytes within one or more body tissues. The ingress of leukocytes into a tissue from the blood stream is directed by the adhesion molecules and chemokines that are expressed on the vascular endothelial lining of blood vessels coursing through that tissue [1]. By recognizing receptors on the leukocyte surface, endothelial proteins coordinate the consecutive stages of leukocyte migration: rolling; firm adhesion; spreading and crawling; and diapedesis. This interaction has specificity, since endothelial adhesion molecules and chemokines differ between vascular beds and across immunopathologies [2, 3].

Targeting the endothelial adhesion molecules that control leukocyte trafficking into a tissue during inflammation has appeal as a biological treatment for inflammatory disease [4]. However, a major challenge in the therapeutic application of any biologic drug is the need to permit normal physiological processes, while simultaneously targeting disease mechanisms. Adhesion molecules also participate in leukocyte migration for immune surveillance, which is the process by which leukocytes patrol the organs of the body, to determine the need for immune responses to threats such as tissue injury, infectious pathogens, and tumor cells [5, 6]. Inhibiting this physiological leukocyte activity could place the patient with inflammatory disease at high risk of infection and/or cancer. Indeed, clinical use of the biologic drug, natalizumab, which targets adhesion molecule, *α*4-integrin (CD49D), has been linked to the occurrence of melanoma, lymphoma, infections, and progressive multifocal leukoencephalopathy [7].

During inflammation, expression of endothelial adhesion molecules and chemokines increases in response to cytokines and other inflammatory mediators [8]. Induction of these proteins depends on the molecular signal(s) and the endothelial subpopulation, and control may be exercised at the level of gene transcription [9]. These observations suggest an innovative therapeutic approach: targeting the induced expression of an endothelial adhesion molecule involved in leukocyte migration might spare constitutive expression and allow physiological functions to continue undisturbed.

Noninfectious posterior uveitis is a group of vision-threatening, retina-based inflammatory diseases mediated by CD4+ helper T cells and myeloid cells [10]. Work from our group and from others has demonstrated a significant role for the immunoglobulin (Ig) superfamily member, intercellular adhesion molecule 1 (ICAM-1, CD54), in interactions between human leukocyte subsets and the retinal vascular endothelium [11, 12]. Master inflammatory cytokine, tumor necrosis factor-alpha (TNF-*α*), plays an important role in the progression of noninfectious posterior uveitis. Examination of blood and ocular fluid samples from affected persons with uveitis indicates high levels of TNF-*α* [13–16], and TNF-*α* blockade is often highly effective in patients with recalcitrant inflammation [17, 18]. In a proof-of-concept study, we investigated the impact of transcription factor blockade on TNF-*α*-induced—versus constitutive—ICAM-1 expression by human retinal endothelial cells and on the interaction between leukocytes and human retinal endothelium.

## 2. Materials and Methods

### 2.1. RNA Silencing Reagents, Cytokines, and Antibodies

Nontargeted small interfering (si)RNA (Silencer Select Negative Control Number 1 siRNA, catalogue number 4390843) and siRNA designed to target nuclear factor of kappa light polypeptide gene enhancer in B-cells 1 (NF-*κ*B1) (Silencer Select siRNA, catalogue number 4392420) were purchased from ThermoFisher Scientific-Ambion (Foster City, CA). Lipofectamine RNAiMAX Transfection Reagent was sourced from ThermoFisher Scientific-Invitrogen (Carlsbad, CA). Human recombinant TNF-*α* was bought from R&D Systems (Minneapolis, MN). Mouse monoclonal anti-human ICAM-1 antibody (clone LB-2, isotype IgG_2b,*κ*_) and mouse monoclonal IgG (clone 27-35, isotype IgG_2b,*κ*_) were obtained from BD Pharmingen (San Jose, CA). Alexa Fluor 488-conjugated goat anti-mouse IgG (H + L) secondary antibody was obtained from ThermoFisher Scientific-Molecular Probes (Eugene, OR).

### 2.2. Endothelial Cell and Leukocyte Lines

Our method for generating human retinal endothelial cell lines has previously been reported in detail [22]. In brief, endothelial cells were isolated from human cadaver retinae by enzymatic digestion of tissue and selection with magnetic bead-conjugated anti-human CD31 antibody and subsequently expanded by transduction with the mouse recombinant amphotropic retrovirus, LXSN16E6E7 [23]. Work presented in our previous publication shows that these retinal endothelial cell lines retain their endothelial phenotype, including expression of endothelial markers and formation of capillary-like tubes on basement membrane substitute [22]. Human retinal endothelial cells were cultured in MCDB-131 medium (Sigma-Aldrich, St. Louis, MO) supplemented with 10% fetal bovine serum (FBS) (HyClone-GE Healthcare Life Sciences, Logan, UT) and endothelial growth factors (EGM-2 SingleQuots supplement, omitting FBS, hydrocortisone, and gentamicin; Clonetics-Lonza, Walkersville, MD) at 37°C and 5% CO_2_. The THP-1 human leukocyte line (American Type Culture Collection, Manassas, VA) was maintained in suspension in RPMI-1640 medium (ThermoFisher-GIBCO, Grand Island, NY) supplemented with 10% FBS and 0.05 mM 2-mercaptoethanol.

### 2.3. RNA Isolation, Reverse Transcription, and Polymerase Chain Reaction

At the conclusion of retinal endothelial cell manipulations, culture medium was replaced with Buffer RLT (Qiagen, Hilden, Germany) with 0.55 mM *β*-mercaptoethanol (Sigma-Aldrich, St. Louis, MO), and cells were frozen at −80°C prior to RNA extraction. Total RNA was extracted using the RNeasy mini kit (Qiagen), according to the manufacturer's instructions and including the optional on-column DNase treatment. RNA concentration was determined by spectrophotometry on the NanoDrop 2000 instrument (ThermoFisher Scientific, Wilmington, DE). Reverse transcription was performed using the iScript Reverse Transcription Supermix for RT-qPCR (Bio-Rad, Hercules, CA), with 250 ng (quantitative real-time polymerase chain reaction (PCR)) or 500 ng (PCR array) of RNA template yielding 20 *μ*L cDNA.

Quantitative real-time PCR was performed on the CFX Connect Real-Time PCR Detection System (Bio-Rad) using 2 *μ*L of cDNA, 4 *μ*L of iQ SYBRGreen Supermix, 1.5 *μ*L each of 20 *μ*M forward and reverse primers, and 11 *μ*L of nuclease-free water for each reaction. Amplification consisted of a precycling hold at 95°C for 5 minutes; 40 cycles of denaturation for 30 seconds at 95°C; annealing for 30 seconds at 60°C; extension for 30 seconds at 72°C; and a postextension hold at 75°C or 81°C for 1 second. A melting curve, consisting of a 1-second hold at every 0.5°C between 70°C and 95°C, was generated to confirm that a single PCR product was produced. For each primer set, standard curves were generated with serially diluted PCR product to confirm an efficiency of 90% or greater. Primer sequences for all transcripts, including reference genes used for normalization, are presented in Table 1.

### 2.4. Polymerase Chain Reaction Array Profiling and Selection of Transcription Factors

Confluent monolayers of human retinal endothelial cells in modified MCDB-131 medium were treated with TNF-*α* (10 ng/mL) or no cytokine (*n* = 5 monolayers/condition) for 60 minutes or 24 hours at 37°C and 5% CO_2_. Total RNA was isolated and reverse-transcribed, as described above. RNA integrity numbers were 9.7 or higher by the 2100 Bioanalyzer (Agilent Technologies, Waldbronn, Germany). Polymerase chain reaction array was performed using the Bio-Rad PrimePCR Transcription Factor (SAB Target List) H96 Assay PCR array and following the manufacturer's instructions exactly, including the use of SsoAdvanced Universal SYBR Green Supermix (Bio-Rad), with 2 *μ*L cDNA per reaction. Results for TNF-*α*-treated versus control cells were normalized to the array reference genes—GAPDH, HPRT1, and TBP—and compared using the “Gene Study” function of CFX Manager software (v.3.1, Bio-Rad).

In order to identify one candidate transcription factor for our proof-of-concept study, we required significant increase from baseline expression at both 60 minutes and 24 hours (*p* < 0.01), plus a 1.5-fold increase in expression at one or both time points. We interrogated the ICAM-1 regulatory sequence for potential binding sites of transcription factors that fulfilled these criteria. We obtained human ICAM-1 transcript sequence from the National Center for Biotechnology Information “Nucleotide” database (i.e., NG_012083.1:* Homo sapiens* intercellular adhesion molecule 1 (ICAM1), RefSeqGene on chromosome 19), and we defined the regulatory sequence as the 5000 bp of sequence immediately upstream of the ICAM-1 gene transcription start site, plus the first 2 introns (3538 bp and 8452 bp). The regulatory sequence was searched for putative binding sites of human transcription factors using the public JASPAR database [21], with relative profile score threshold set at 80%.

### 2.5. RNA Silencing

Confluent monolayers of human retinal endothelial cells were transfected with 10 nM NF-*κ*B1-targeted siRNA or nontargeted control siRNA using 1.7 *μ*L/mL Lipofectamine RNAiMAX Reagent in modified MCDB-131 medium, following the manufacturer's “Forward Transfection” protocol for human umbilical vein endothelial cells. Concentrations, timing, and siRNA were determined in a pilot study (data not shown). After 24 hours, the transfection mixture was replaced with medium only. Monolayers treated with NF-*κ*B1-targeted siRNA or nontargeted siRNA were cultured for an additional 24 hours and subsequently treated with TNF-*α* (10 ng/mL) or no cytokine in fresh medium for a final 24 hours.

### 2.6. Retinal Endothelial ICAM-1 ELISA

The human retinal endothelial ICAM-1 ELISA was adapted from the protocol published by Hartwig et al. [24]. Confluent monolayers of endothelial cells in wells of 96-well plates, transfected with NF-*κ*B1-targeted siRNA or nontargeted control siRNA and treated with TNF-*α* or no cytokine, were washed twice in phosphate buffered saline (PBS) with 0.1% Tween-20 (PBS/Tween-20), fixed in 1% paraformaldehyde for 30 minutes, and washed in PBS/Tween-20. Following a 30-minute block with 5% w/v skim milk in PBS (blocking solution), monolayers were incubated with anti-human ICAM-1 antibody or mouse monoclonal IgG at 1 *μ*g/mL in blocking solution for 45 minutes at room temperature (*n* = 8 monolayers/condition) and washed 5 times with PBS/Tween-20. Subsequently, monolayers were incubated with Alexa Fluor 488-conjugated secondary antibody at 2.5 *μ*g/mL in blocking solution for 30 minutes at room temperature and washed 5 times with PBS/Tween-20. Finally monolayers were treated with 300 nM 4′,6-diamidino-2-phenylindole-dihydrochloride (DAPI) (Sigma-Aldrich) in PBS for 5 minutes and washed 3 times in PBS. All treatments after the fixation step were performed on a slowly rotating orbital shaker.

Monolayer fluorescence was determined on the VICTOR X3 Multilabel Plate Reader (Perkin Elmer, Waltham, MA) using 485 excitation and 535 emission (Alexa Fluor 488) filters and 355 excitation and 460 emission (DAPI) filters. Mean background fluorescence was determined from mouse IgG-incubated wells for each condition, and this value was subtracted from wells incubated with anti-human ICAM-1 antibody, for the matching condition. Any difference in cell numbers between wells was accounted for by adjusting Alexa Fluor 488 readings for DAPI readings from the same wells.

### 2.7. Leukocyte-Retinal Endothelial Cell Adhesion Assay

Confluent monolayers of human retinal endothelial cells in wells of 48-well plates, transfected with NF-*κ*B1-targeted siRNA or nontargeted control siRNA and treated with TNF-*α* or no cytokine, were incubated with 1 × 10^5^ THP-1 leukocytes suspended in modified MCDB-131 medium (*n* = 5 monolayers/condition) for 20 minutes at 37°C and 5% CO_2_. After removal of the THP-1 leukocyte suspensions, monolayers were washed 4 times in medium, rotating the plate 90 degrees with each wash, and fixed in 10% neutral buffered formalin for 10 minutes. The center of each well was photographed under 100x magnification on the IX53 inverted microscope (Olympus, Tokyo, Japan) and with a UC50 color CCD camera (Olympus Soft Imaging Solutions, Muenster, Germany) and cellSens Imaging Software (v.1.8.1, Olympus). The number of THP-1 leukocytes bound to the human retinal endothelial cell monolayer in each photograph was counted and converted to bound leukocytes/mm.

### 2.8. Statistical Analysis

Analysis of the PCR array is described above. For other experiments, data for test and control conditions were compared by two-tailed Student's *t*-tests, using GraphPad Prism v6.04 (GraphPad Software, La Jolla, CA). In all analyses, a significant difference was defined as one yielding a *p* value less than 0.05.

## 3. Results and Discussion

**Figure 1:**
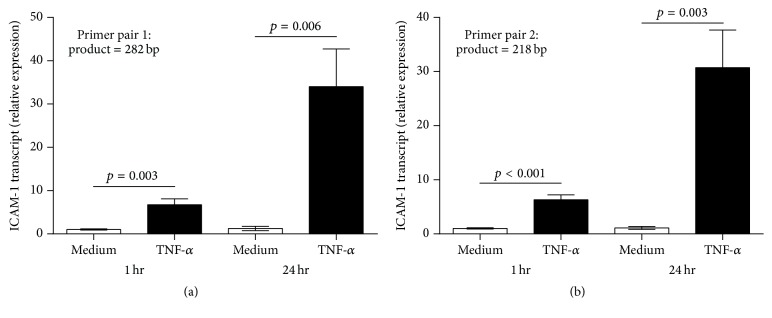
Graphs showing relative ICAM-1 transcript expression in human retinal endothelial cell monolayers, following stimulation with TNF-*α* for 60 minutes or 24 hours, as determined by qRT-PCR using two ICAM-1 primer pairs. Reference gene was 18S rRNA. The same result was obtained when data were normalized to cyclophilin A reference gene. (a) shows the result obtained using ICAM-1 primer pair listed in Table 1 that yields 282 bp product. (b) shows similar result obtained using ICAM-1 primer pair listed in Table 1 that yields 218 bp product. Bars represent mean relative expression, with error bars showing standard error of the mean. *n* = 5 monolayers/condition. Data were analyzed by two-tailed Student's *t*-test. Human retinal endothelial RNA samples used for this experiment were used for PCR array profiling study presented in Table 2.

Our proof-of-concept study explored the effect of transcription factor blockade on induced—versus constitutive—expression of an endothelial adhesion molecule in a human uveitis model. Noninfectious posterior uveitis involves the migration of multiple leukocyte subsets across the retinal vascular endothelium. Intercellular adhesion molecule-1 is a key adhesion molecule for leukocyte egress into human retina [11, 12], and TNF-*α* is a master cytokine regulator of inflammation within human retina [13–16]. We treated human retinal endothelial cells with TNF-*α* and observed a substantial increase in ICAM-1 transcript expression over 24 hours, beginning within 60 minutes of exposure (Figure 1). This finding indicates that* ICAM-1* gene transcription might be targeted to limit induction of ICAM-1 protein expression on retinal endothelial cells and leukocyte-retinal endothelial interactions, without impacting baseline levels of these parameters.

Expression of ICAM-1 is regulated primarily at transcription; activation of the* ICAM-1* gene regulatory sequence is cell- and stimulus-specific [25, 26]. To identify transcription factors involved in retinal endothelial ICAM-1 induction, we profiled TNF-*α*-treated human retinal endothelial cells against cytokine-naïve control cells, using a PCR array that detected 86 well-characterized human transcription factors. In analyzing the results of the PCR array, we focused on transcription factors that increased significantly in parallel with ICAM-1 and demonstrated at least 1.5-fold increase in expression, following exposure to TNF-*α*. The 5000 bp of sequence immediately upstream of the* ICAM-1* gene transcription start site, plus the first 2 introns (3538 bp and 8452 bp), were searched for putative binding sites of the candidate transcription factors. We employed JASPAR, which is an open-access database of matrix-based nucleotide transcription factor binding profiles [21].

Seven transcription factors were upregulated in human retinal endothelial cells on exposure to TNF-*α* and had potential binding sites in the* ICAM-1* regulatory sequence (Table 2). For the purposes of proving concept, we selected NF-*κ*B1 from the group of 7 candidate transcription factors. This selection was based on the knowledge that NF-*κ*B1 has been linked to noninfectious posterior uveitis; for example, several groups have reported an association between* NF-κB1* polymorphisms and Behçet disease, in which posterior uveitis is classically an occlusive retinal vasculitis [27, 28]. The JASPAR analysis indicated that the* ICAM-1* regulatory region included at least 25 putative binding sites for NF-*κ*B1 (Table 3).

Despite initial enthusiasm for therapeutic manipulation of gene expression, transcription soon came to be regarded as “undruggable” [29]. Potential targets in transcription include (1) factors that bind specific DNA sequences to activate or inhibit RNA polymerase II-controlled transcription; (2) coregulators that bind transcription factors to enhance or repress activity; and (3) DNA methylation and histone modifications that direct access of transcription factors to DNA-binding sites. However, the challenges to targeting are significant and include the multiple gene targets of each transcription factor; predominant nuclear location; and large surface areas for DNA- and protein-protein interactions. Over the past 5 years, multiple advances have begun to address these concerns, particularly in oncology, where transcription factor-targeted drugs are now in clinical trials [30]. DNA-binding small molecules, polyamides, and oligonucleotide-based decoys inhibit binding of transcription factors to promoters [29]. Transcription activator-like effectors (TALEs) and zinc finger proteins (ZFPs) provide the opportunity to target specific DNA sequences [31].

**Figure 2:**
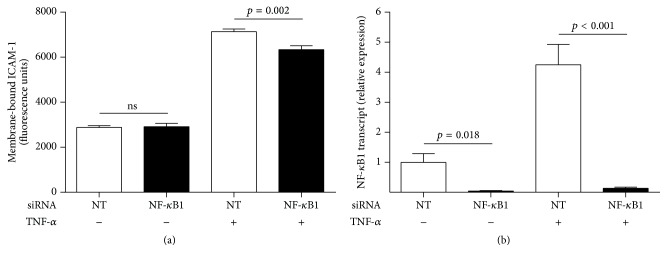
TNF-*α*-induced expression of cellular ICAM-1 is significantly reduced by NF-*κ*B1 silencing, but constitutive expression is not impacted. Human retinal endothelial cell monolayers were transfected with NF-*κ*B1-targeted (NF-*κ*B1) siRNA or nontargeted (NT) control siRNA and treated with TNF-*α* or no cytokine for 24 hours. Cellular ICAM-1 was fluorescently labeled by indirect immunocytochemistry; fluorescence of endothelial monolayers was read by microplate reader and adjusted for background fluorescence and cell number. (a) Graph showing relative level of ICAM-1 protein on the surface of endothelial cells. Bars represent mean relative level, with error bars showing standard error of the mean. *n* = 8 monolayers/condition. Data were analyzed by two-tailed Student's *t*-test. ns = not significant. (b) Graph showing relative NF-*κ*B1 transcript expression in human retinal endothelial cell monolayers that were transfected in parallel. Reference gene was 18S rRNA. Bars represent mean relative expression, with error bars showing standard error of the mean. *n* = 4 monolayers/condition. Data were analyzed by two-tailed Student's *t*-test.

**Figure 3:**
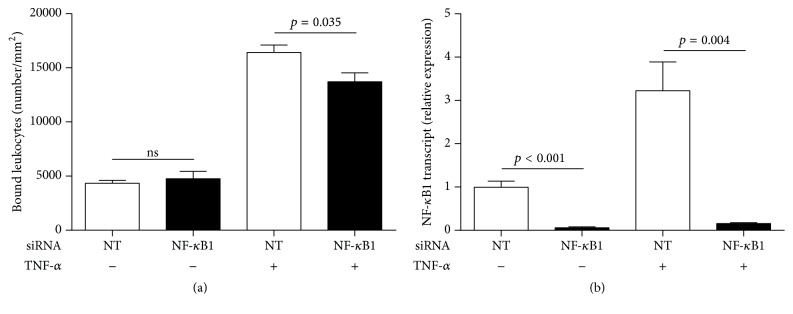
Leukocyte binding to TNF-*α*-stimulated human retinal endothelial monolayers is significantly reduced by NF-*κ*B1 silencing, but binding to untreated monolayers is not impacted. Human retinal endothelial cell monolayers were transfected with NF-*κ*B1-targeted (NF-*κ*B1) siRNA or nontargeted (NT) control siRNA and treated with TNF-*α* or no cytokine for 24 hours. Endothelial monolayers were incubated with THP-1 leukocytes for 20 minutes (1 × 10^5^ cells/7.5 mm^2^ monolayer); monolayers were photographed, and the number of leukocytes bound per mm^2^ was counted. (a) Graph showing leukocytes bound to the surface of endothelial monolayers. Bars represent mean number, with error bars showing standard error of the mean. *n* = 5 monolayers/condition. Data were analyzed by two-tailed Student's *t*-test. ns = not significant. (b) Graph showing relative NF-*κ*B1 transcript expression in human retinal endothelial cell monolayers that were transfected in parallel. Reference gene was 18S rRNA. Bars represent mean relative expression, with error bars showing standard error of the mean. *n* = 4 monolayers/condition. Data were analyzed by two-tailed Student's *t*-test.

We silenced NF-*κ*B1 transcript with siRNA to examine the effect of targeting TNF-*α*-induced* ICAM-1* gene transcription in human retinal endothelial cells. ICAM-1 exists as both soluble and membrane-bound forms; only the membrane-bound form is relevant to the leukocyte-retinal endothelial cell interaction [1]. Thus, we measured the effect of NF-*κ*B1 silencing on human retinal endothelial cell surface ICAM-1 expression, using a cellular ELISA. Knockdown of NF-*κ*B1 significantly decreased TNF-*α*-induced ICAM-1 protein expression but did not reduce constitutive ICAM-1 expression (Figure 2). We next examined the effect of NF-*κ*B1 silencing on leukocyte-endothelial cell interactions, by quantifying THP-1 leukocyte binding to human retinal endothelial monolayers. Knockdown of NF-*κ*B1 significantly decreased leukocyte binding to human retinal endothelial monolayers that were pretreated with TNF-*α* but did not reduce binding to nontreated monolayers (Figure 3). Thus, NF-*κ*B1-targeted blockade of* ICAM-1* gene transcription reduces TNF-*α*-induced inflammatory activities of human retinal endothelial cells that contribute to the development of noninfectious posterior uveitis. Importantly, the blockade does not impact baseline levels of these activities.

## 4. Conclusions

Our research explores a novel treatment paradigm for noninfectious posterior uveitis that also has application to human inflammatory diseases outside the retina. We show the feasibility of targeting induced—but not constitutive—expression on the endothelium of a key adhesion molecule involved in initiation of an inflammatory disease. Sparing constitutive expression is expected to limit the side effects that have complicated the use of other biologic drugs due to the roles of target molecules in physiological leukocyte activity. Targeting transcription of endothelial adhesion molecules might result in changes within the cell that lead to other complications. However, current technologies permit highly specific transcription blockade, and recent progress within the pharmaceutical field promises the possibility of local delivery to many organs. Important tasks in translating this therapeutic approach to the clinic are the need to define tissue- and disease-specific adhesion molecules for a given inflammatory condition and consideration of individual utilization of those adhesion molecules in leukocyte-endothelial interactions across multiple human subjects.

## Figures and Tables

**Table 1:** Primer pairs and product sizes for gene transcripts studied in human retinal endothelial cells. References are provided for primer sequences sourced from the literature. For primer pairs designed in-house, products were confirmed by sequencing.

Gene transcript^*∗*^	Primer pair	Product size (bp)
NF-*κ*B1 [19]	Forward: 5′-CCCAGTGAAGACCACCTCTC-3′	132
	Reverse: 5′-CTGAGTTTGCGGAAGGATGT-3′	

ICAM-1 [20]	Forward: 5′-TAAGCCAAGAGGAAGGAGCA-3′	282
	Reverse: 5′-CATATCATCAAGGGTTGGGG-3′	

ICAM-1	Forward: 5′-GGCCTCAGTCAGTGTGA-3′	218
	Reverse: 5′-AACCCCATTCAGCGTCA-3′	

18S rRNA	Forward: 5′-GTAACCCGTTGAACCCCATT-3′	151
	Reverse: 5′-CCATCCAATCGGTAGTAGCG-3′	

Cyclophilin A	Forward: 5′-GACCTCTGGAGAGAAAGGATTT-3′	355
	Reverse: 5′-GGTGATCTTCTTGCTGGTCTT-3′	

^*∗*^NF-*κ*B1 = nuclear factor *κ*-light-chain-enhancer of activated B-cells 1; ICAM-1 = intercellular adhesion molecule 1; 18S rRNA = 18S ribosomal RNA.

**Table 2:** Transcription factors with putative binding sites within the regulatory region of intercellular adhesion molecule 1 (*ICAM-1*) that increase in human retinal endothelial cells treated with tumor necrosis factor-alpha (TNF-*α*). For the purposes of this analysis, the *ICAM-1* regulatory region was defined as 5000 bp of sequence immediately upstream of the *ICAM-1* gene transcription start site and the first 2 introns. The *ICAM-1* gene sequence was interrogated in the JASPAR database [21].

Transcription factor^*∗*^	Induction with TNF-*α*				Number of binding sites
	60 minutes		24 hours		
	Fold increase	*p* value	Fold increase	*p* value	
ETS1	1.65	<10^−5^	2.15	<10^−5^	48
EGR1	1.36	<10^−5^	2.31	<10^−5^	268
JunB	2.95	<10^−5^	5.73	<10^−5^	148
IRF1	4.77	<10^−5^	5.11	<10^−5^	83
NF-*κ*B1	1.24	<10^−3^	2.75	<10^−5^	25
c-Rel	1.55	<10^−3^	1.38	<10^−4^	101
TGIF1	1.31	<10^−4^	1.70	<10^−5^	6

^*∗*^ETS1 = E26 transformation-specific 1; EGR1 = early growth response 1; IRF1 = interferon regulatory factor 1; NF-*κ*B1 = nuclear factor *κ*-light-chain-enhancer of activated B-cells 1; TGIF1 = TGFB-induced factor homeobox 1.

**Table 3:** Sequences and locations of NF-*κ*B1 putative binding sites within the regulatory region of intercellular adhesion molecule 1 (*ICAM-1*). For the purposes of this study, the *ICAM-1* regulatory region was defined as 5000 bp of sequence immediately upstream of the *ICAM-1* gene transcription start site and the first 2 introns. Gene sequences were obtained from the JASPAR database [21].

Sequence	Location			
	Region	Start	End	Strand
AGGAGAATCACCT	5000 bp upstream of transcription start site	282	294	−1
AGGTGATTCTCCT		282	294	1
TGGGGTTTCACCA		3008	3020	−1
AGGGAGACCCCCA		3524	3536	−1
TGGGGGTCTCCCT		3524	3536	1
GGGGGACGCCCCT		3874	3886	−1
AGGGGCGTCCCCC		3874	3886	1
GAGGGATGCCCCT		4751	4763	−1
AGGGGCATCCCTC		4751	4763	1

TGGGGATTGCCGT	Intron 1 (3538 bp)	12	24	1
GGGGGAATTCCAG		584	596	1

CGGGGTTTCACCA	Intron 2 (8452 bp)	1333	1345	−1
TGGTGAAACCCCG		1333	1345	1
CGGGGACTTCCCT		3433	3445	−1
AGGGAAGTCCCCG		3433	3445	1
GGGTGGATCACCA		3748	3760	−1
TGGTGATCCACCC		3748	3760	1
CGGTGAAACCCCG		6035	6047	−1
CGGGGTTTCACCG		6035	6047	1
AGGCGGATCACCT		6948	6960	−1
AGGTGATCCGCCT		6948	6960	1
AGGGAGACCCCCA		7545	7557	−1
TGGGGGTCTCCCT		7545	7557	1
AGGGGATTCTCCT		8082	8094	−1
AGGAGAATCCCCT		8082	8094	1
